# Association of Preoperative Prognostic Nutritional Index with Risk of Postoperative Acute Kidney Injury: A Meta-Analysis of Observational Studies

**DOI:** 10.3390/nu15132929

**Published:** 2023-06-28

**Authors:** Chien-Cheng Liu, Ping-Hsin Liu, Hsiao-Tien Chen, Jui-Yi Chen, Chia-Wei Lee, Wan-Jung Cheng, Jen-Yin Chen, Kuo-Chuan Hung

**Affiliations:** 1 Department of Anesthesiology, E-Da Hospital, I-Shou University, Kaohsiung City 82445, Taiwan; jasperliu1552@gmail.com; 2 Department of Nursing, College of Medicine, I-Shou University, Kaohsiung City 82445, Taiwan; 3 School of Medicine, I-Shou University, Kaohsiung City 82445, Taiwan; 4 Department of Anesthesiology, E-Da Dachang Hospital, I-Shou University, Kaohsiung City 82445, Taiwan; neoplasmboy@yahoo.com.tw; 5 Department of Chinese Medicine, Chi Mei Medical Center, Tainan City 71004, Taiwan; sanra770211@gmail.com; 6 Division of Nephrology, Department of Internal Medicine, Chi Mei Medical Center, Tainan City 71004, Taiwan; kwuilus0101@gmail.com; 7 Department of Health and Nutrition, Chia Nan University of Pharmacy and Science, Tainan City 71710, Taiwan; 8 Department of Neurology, Chi Mei Medical Center, Tainan City 71004, Taiwan; kimo1257@gmail.com; 9 Department of Anesthesiology, Chi Mei Medical Center, Liouying, Tainan City 73657, Taiwan; kort1105@hotmail.com; 10 Department of Anesthesiology, Chi Mei Medical Center, Tainan City 71004, Taiwan; chenjenyin@gmail.com; 11 School of Medicine, College of Medicine, National Sun Yat-Sen University, Kaohsiung City 80424, Taiwan

**Keywords:** prognostic nutritional index, acute kidney injury, postoperative, surgery, mortality

## Abstract

This meta-analysis aimed to assess the clinical association of the preoperative prognostic nutritional index (pre-PNI) with the risk of postoperative acute kidney injury. Four databases (e.g., Medline) were searched from inception to December 2022 to investigate the association between pre-PNI (i.e., low vs. high) and PO-PNI as well as the correlation between pre-PNI and other postoperative prognostic indices. Overall, 13 observational studies, including 9185 patients, were eligible for analysis. A low PNI was related to increased risks of PO-AKI [odd ratio (OR) = 1.65, *p* = 0.001, 3811 patients], postoperative infection (OR = 2.1, *p* < 0.00001, 2291 patients), and mortality (OR = 1.93, *p* < 0.0001, 2159 patients). Albeit statistically nonsignificant, a trend was noted, linking a low PNI to higher risks of postoperative bleeding (OR = 2.5, *p* = 0.12, 1157 patients) and stroke (OR = 1.62, *p* = 0.07, 2036 patients). Pooled results revealed a prolonged intensive care unit (ICU) stay in patients with low PNIs compared to those with high PNIs (MD: 0.98 days, *p* = 0.02, 2209 patients) without a difference in hospital stay between the two groups (MD: 1.58 days, *p* = 0.35, 2249 patients). This meta-analysis demonstrated an inverse correlation between PNI and the risks of PO-AKI, postoperative infection, and mortality, as well as the length of ICU stay, which warrants further investigations for verification.

## 1. Introduction

Postoperative acute kidney injury (PO-AKI) is a complication that developed in approximately 9–12% of patients that underwent major surgery [1,2,3]. PO-AKI could be considered a sentinel surgical morbidity because it is strongly associated with several adverse outcomes, including the development of chronic kidney disease (CKD), the co-occurrence of other postoperative complications, a prolonged hospital stay, and an increased risk of death [1,4,5]. However, the causes of PO-AKI are complicated and multifactorial. A number of well-known risk factors are identified, such as old age, pre-existing kidney dysfunction, diabetes, and sepsis [4]. Unfortunately, there is no effective prophylaxis against PO-AKI [6,7]. Therefore, a parameter for early identification of patients at high risk could allow modification of risk factors to minimize the development of this condition.

Many studies have shown a close correlation between a patient’s preoperative inflammatory and nutritional status and both short- and long-term surgical morbidity [8,9]. Not only is serum albumin widely used as a measure of nutritional status, but it is also a protein that regulates renal blood supply by modulating fluid shifts between different body compartments through its role as a determinant of plasma oncotic pressure [10,11]. In addition, because albumin has the ability to bind toxic agents and scavenge free radicals, it has both antioxidative and anti-inflammatory properties [12,13]. Therefore, preoperative serum albumin concentration is considered a better surgical prognostic predictor compared to other preoperative factors [14]. The lymphocyte is one subtype of white blood cells that plays an essential role in inflammation. Surgical stress induces an immune response in which lymphocytes and other anti-inflammatory factors play a vital role. Indeed, a low lymphocyte count has been shown to be predictive of poor survival after surgery [12,15].

The prognostic nutritional index (PNI) is an objective and convenient biological marker that reflects a patient’s status of nutrition and immunity by calculating the serum albumin levels and total lymphocyte counts in peripheral blood [16]. Although PNI appears to be associated with survival and complications after surgery [17,18,19], there are few studies on the association between preoperative PNI and PO-AKI in surgical patients. Therefore, the aim of this systematic review and meta-analysis was to evaluate the association between pre-PNI and PO-AKI in patients undergoing major surgery.

## 2. Materials and Methods

### 2.1. Protocol Registration

We complied using the Preferred Reporting Items for Systematic Reviews and Meta-Analyses Statement (PRISMA) guidelines when reporting the current meta-analysis (PROSPERO CRD42022332714), in which two independent reviewers selected the study, collected data, and assessed the quality of studies. A third reviewer was consulted for any disagreements between the two reviewers.

### 2.2. Search Strategies and Databases

We searched four data sources, namely Google Scholar, Embase, Medline, and the Cochrane Library, to identify published articles investigating the association of preoperative PNI with PO-AKI risk from inception to 21 December 2022. Boolean operators “AND” or “OR” were applied to combine different search terms to narrow down or expand the results of a search. The following keywords were used for literature searches: (“Postoperative” or “Surger*” or “Surgical Procedure*” or “Operative Procedur*s” or “General anesthesia” or “Operation” or “Surgical” or “Cardiac Surgical Procedure*” or “Open heart surger*” or “Valvular heart surger*”) and (“Prognostic nutritional index” or “Prognostic Nutritional Indices” or “PNI”), and (“Acute kidney injury” or “AKI” or “Acute renal failure” or “Kidney injury” or “Acute Kidney Insufficiency” or “Kidney Tubular Necrosis” or “Renal Insufficiency”). Subject headings, such as mesh terms, were also used to assist our database search (Supplemental Table S1). We also checked references of the review articles and relevant studies to identify other studies that may be relevant to our research. There were no restrictions on publication year, type of surgery, geographical regions, language, or sample size. For any missing information in the article, the corresponding author was contacted three times.

### 2.3. Criteria for Inclusion and Exclusion of Studies

The following criteria were applied in the selection of eligible studies: (1) randomized controlled studies or observational cohort studies, (2) adult patients undergoing surgery regardless of procedures, (3) available preoperative PNI before surgical intervention, (4) reporting of the relationship between preoperative PNI and PO-AKI risk, and (5) studies with available data (e.g., events/total number of cases) for the calculation of effect size [e.g., odds ratio (OR)].

We excluded studies that (1) recruited pediatric population or patients not receiving surgical interventions, (2) were presented as conference abstracts, case reports, review articles, or (3) were not peer-reviewed publications.

### 2.4. Extraction of Data and Study Outcomes

For each study, two authors independently extracted details regarding patient characteristics (i.e., age, gender distribution, and number of patients), preoperative creatinine levels, incidence of patients with chronic kidney disease, author information (i.e., first author’s name and country), type of surgery, definition of PO-AKI, and relevant outcomes (i.e., incidence of PO-AKI and PNI values). Discrepancy between data collection was resolved through discussion. The association of preoperative PNI with the risk of PO-AKI, which was defined based on that of each study, was set as the primary outcome. The secondary outcomes included the correlation between preoperative PNI and other postoperative prognosis factors, namely the risk of infection, mortality, bleeding, and stroke, as well as hospital/intensive care unit (ICU) length of stay (LOS).

### 2.5. Quality of Studies and Certainty of Evidence

We used the Newcastle-Ottawa Scale (NOS) and ROB 2.0 to assess the quality of observational cohort studies and randomized controlled studies, respectively. Retrospective studies were considered to be of higher quality if more than seven stars on the NOS were assigned. Two independent authors judged the certainty of evidence by categorizing each outcome into one of four grades (i.e., high, moderate, low, and very low). Disagreements regarding judgment on certainty of evidence were settled through consensus.

### 2.6. Statistical Methods for the Analysis

A random-effects model was used to calculate the pooled odds ratios (ORs)/mean difference (MD). For each outcome, the 95% confidence intervals (CIs) were also reported. The heterogeneity between studies was computed using *I*^2^ statistics and classified into three categories, namely low (0% to 50%), moderate (51% to 75%), and high (76% to 100%). The robustness of outcomes was assessed using leave-one-out sensitivity analysis by removing one dataset at a time, while publication bias was investigated by inspecting funnel plots if more than 10 datasets were available. Subgroup analysis based on the surgical approach (i.e., cardiac vs. noncardiac surgery) was performed. All statistical analyses were conducted with Review Manager (RevMan 5.3; Copenhagen, Denmark: The Nordic Cochrane Centre, The Cochrane Collaboration, 2014) and the comprehensive Meta-Analysis (CMA) V3 software (Biostat, Englewood, NJ, USA). A probability value less than 0.05 was set for all analyses as a threshold for statistical significance.

## 3. Results

### 3.1. Characteristics and Quality of Studies

**Figure 1:**
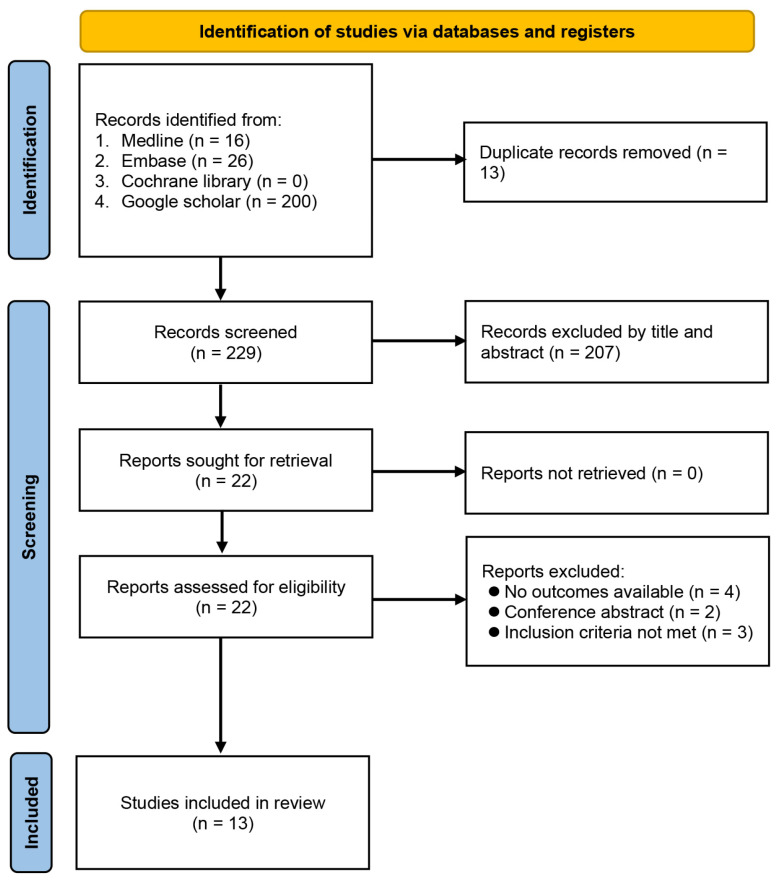
Flowchart of study selection.

The process of study identification from database searches is demonstrated in Figure 1. Initially, we identified 242 records, of which 220 were removed because they were duplicate publications (*n* = 13) or did not meet the selection criteria (*n* = 207). Full-text review was independently performed by two authors on the remaining 22 publications, of which 13 met our predefined criteria for inclusion. The 13 eligible articles were retrospective studies published between 2019 and 2022, including a total of 9185 patients [12,17,19,20,21,22,23,24,25,26,27,28,29].

**Figure 2:**
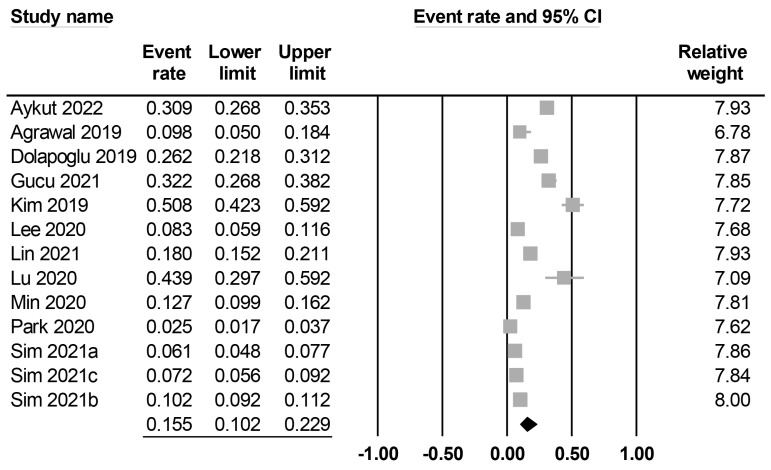
Forest plot showing pooled incidence of postoperative acute kidney injury [12,17,19,20,21,22,23,24,25,26,27,28,29], The studies’ effect sizes are represented by squares, while the pooled effect is depicted as a diamond.

The characteristics of the included studies are summarized in Table 1. All studies included middle-aged patients (mean or median age: 44 to 69 years), with male proportions ranging between 56.8% and 88%. Twelve studies reported details on body mass index (i.e., 20–31 kg/m^2^), while this information was unavailable in one study [20]. Among the included studies, seven explicitly reported the exclusion of patients with chronic kidney disease [12,20,22,25,27,28,29], whereas five did not specify whether such exclusions were made [17,19,21,23,26]. Contrarily, one study reported a chronic kidney disease incidence of 5.6% among the study population [24]. Preoperative creatinine levels were available in nine studies (Supplemental Table S2) [12,19,21,22,23,25,26,28,29], whereas five studies did not provide this information. There was a wide variation in sample size among the studies, with a range of 41–3543. The 13 included studies focused on three major surgical approaches, including cardiac surgery (*n* = 5) [12,21,22,24,25], abdominal surgery (*n* = 5) [19,20,27,28,29], and lung surgery (*n* = 3) [17,23,26]. Four studies reported surgical procedures were performed electively [12,19,28,29], while other studies did not specify this information. In all the included studies, the prognostic nutritional index (PNI) was calculated using the formula [10 × serum albumin (g/dL)] + [0.005 × total lymphocyte count (count/mm^3^)]. The cut-off values of PNI were available in eight studies [17,19,20,21,23,24,25,26], while they were not mentioned in five studies [12,22,27,28,29]. The definition of PO-AKI was summarized in Supplemental Table S2. Among the included studies, five used the Kidney Disease Improving Global Outcomes (KDIGO) classification (stages I–III) [19,21,22,28,29] and two used the Acute Kidney Injury Network (AKIN) classification (stages II–III) [17,27] to define the occurrence of PO-AKI. Furthermore, five studies defined PO-AKI based on an increase in serum creatinine from baseline, such as a minimum increase of 0.3 mg/dL or a twofold elevation in creatinine level compared with preoperative values [12,23,24,25,26]. However, one study did not provide specific information regarding the PO-AKI definition used [20]. The incidence of PO-AKI was reported in all studies (ranging between 2.5% and 50.8%), with a pooled incidence of 15.5% (95% CI: 10.2% to 22.9%) (Figure 2). Studies were conducted in four countries, including India (*n* = 1) [20], China (*n* = 2) [25,26], Turkey (*n* = 3) [12,21,22], and Korea (*n* = 7) [17,19,23,24,27,28,29]. The quality of the studies is summarized in Table 1. Of 13 studies, one study was considered low quality (i.e., NOS < 7) [29], while the other 12 studies were considered high quality.

### 3.2. Outcomes and Certainty of Evidence

#### 3.2.1. Primary Outcomes

**Figure 3:**
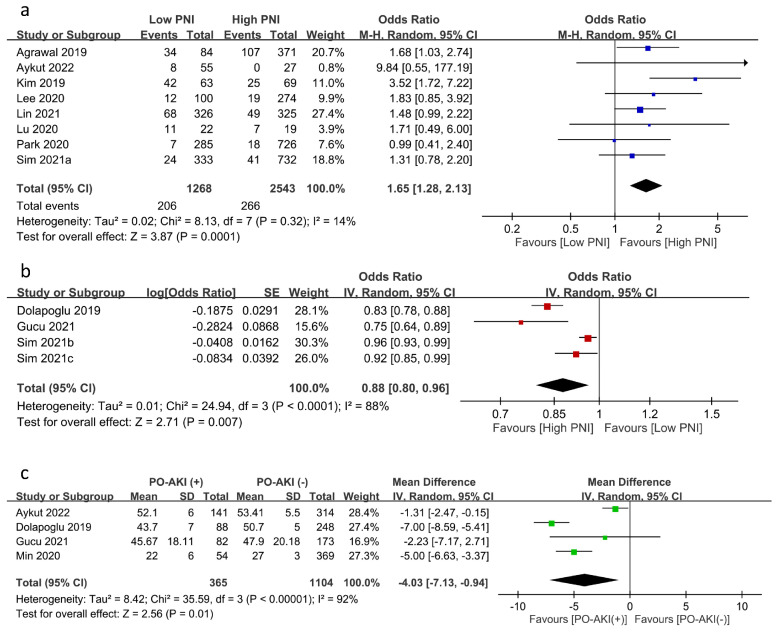
Forest plot showing (**a**) the risk of postoperative acute kidney injury (PO-AKI) in patients with a low or high prognostic nutritional index (PNI) [17,19,20,21,23,24,25,26], (**b**) the association of PO-AKI risk with PNI value as a continuous variable [12,22,28,29], and (**c**) the difference in PNI values in patients with or without PO-AKI [12,21,22,27]. CI: confidence interval; M–H: Mantel–Haenszel; IV: inverse variance; SD: standard deviation; SE: standard error. The studies’ effect sizes are represented by squares, while the pooled effect is depicted as a diamond.

The relationship between PNI and the risk of PO-AKI was reported using PNI as a categorical variable (i.e., low PNI vs. high PNI groups) (eight studies) [17,19,20,21,23,24,25,26] or a continuous variable (four studies) [12,22,28,29]. Meta-analysis of eight studies focusing on PNI as a category variable revealed an inverse association of low PNI with PO-AKI risk (OR = 1.65, 95% CI: 1.28 to 2.13, *p* = 0.001, *I*^2^:14%, eight studies, 3811 patients) (Figure 3a) [17,19,20,21,23,24,25,26]. Sensitivity analysis showed a consistent finding, which supported the robustness of this outcome. Similarly, using PNI as a continuous variable demonstrated an inverse correlation between PNI and the risk of PO-AKI (OR: 0.877, 95% CI: 0.798 to 0.965, *p* = 0.007, *I*^2^ = 88%) (Figure 3b) [12,22,28,29] with a consistent finding on sensitivity analysis. In addition, the PNI values in patients with or without AKI were available in four studies, revealing significantly lower PNI values in patients with PO-AKI (MD: −4.03 95%CI: −7.13 to −0.94, *p* = 0.01, *I*^2^ = 92%, four studies, 1469 patients) (Figure 3c) [12,21,22,27]. Sensitivity analysis using the leave-one-out approach demonstrated an inconsistent finding.

#### 3.2.2. Secondary Outcomes

**Figure 4:**
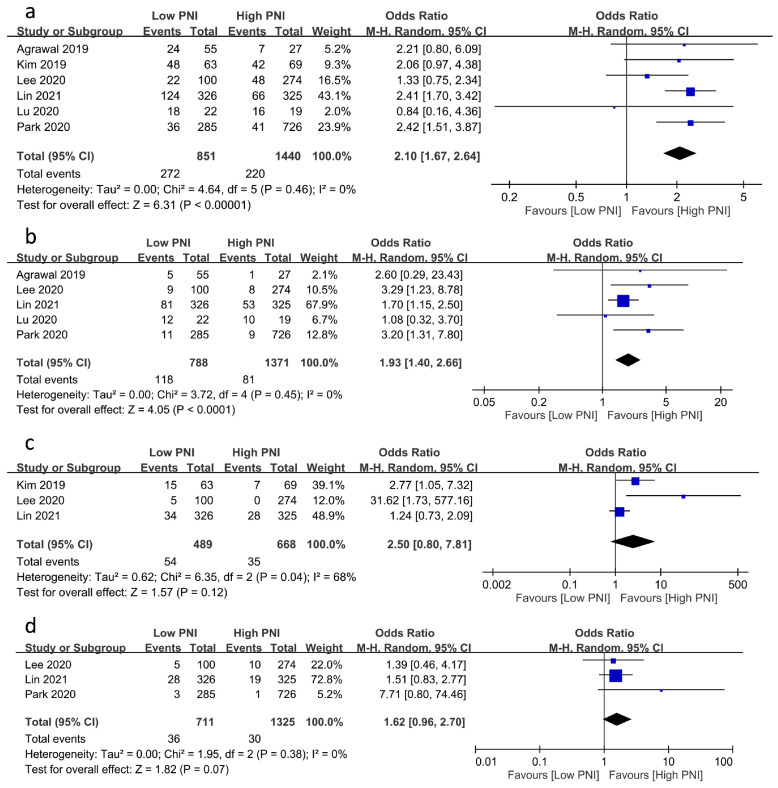
Forest plot showing the association of low prognostic nutritional index (PNI) values with postoperative risk of (**a**) infection [20,23,24,25,26], (**b**) mortality [17,20,24,25,26], (**c**) bleeding [17,18,19], and (**d**) stroke [17,24,25]. CI: confidence interval; M–H: Mantel–Haenszel. The studies’ effect sizes are represented by squares, while the pooled effect is depicted as a diamond.

The associations of PNI with the risks of other postoperative complications are demonstrated in Figure 4. A low PNI was related to an increased risk of postoperative infection (OR = 2.1, 95% CI: 1.67 to 2.64, *p* < 0.00001, *I*^2^ = 0%, six studies, 2291 patients, sensitivity analysis: consistent) (Figure 4a) [20,23,24,25,26] and mortality (OR = 1.93, 95% CI: 1.4 to 2.66, *p* < 0.0001, *I*^2^ = 0%, five studies, 2159 patients, sensitivity analysis: consistent) (Figure 4b) [17,20,24,25,26]. Despite the lack of a significant correlation between PNI and the risks of postoperative bleeding and stroke, a low PNI tended to be associated with a higher risk of postoperative bleeding (OR = 2.5, 95% CI: 0.8 to 7.81, *p* = 0.12, *I*^2^ = 68%, three studies, 1157 patients, sensitivity analysis: consistent) (Figure 4c) [17,18,19] and stroke (OR = 1.62, 95% CI: 0.96 to 2.7, *p* = 0.07, *I*^2^ = 0%, three studies, 2036 patients, sensitivity analysis: consistent) (Figure 4d) [17,24,25].

**Figure 5:**
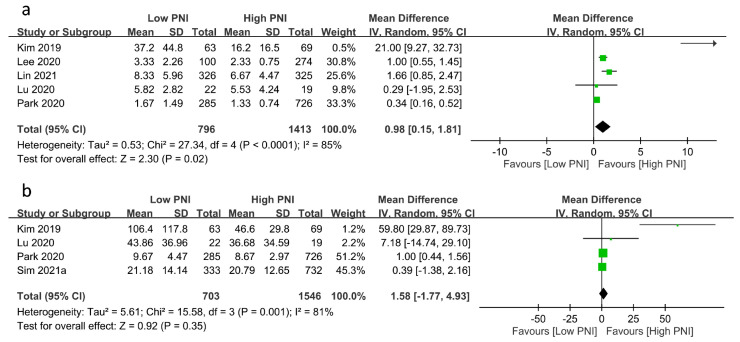
Forest plot demonstrating the association of a low prognostic nutritional index (PNI) with (**a**) intensive care unit stay [23,24,25,26], and (**b**) hospital stay [17,19,23,26]. CI: confidence interval; IV: inverse variance; SD: standard deviation. The studies’ effect sizes are represented by squares, while the pooled effect is depicted as a diamond.

The relationships between PNI and medical resource utilization are shown in Figure 5. Pooled results revealed a prolonged ICU stay in patients with a low PNI compared to those with a high PNI (MD: 0.98 days, 95% CI: 0.15 to 1.81, *p* = 0.02, *I*^2^ = 85%, five studies, 2209 patients, sensitivity analysis: inconsistent) (Figure 5a) [23,24,25,26]. However, there was no difference in hospital stay between the two groups (MD: 1.58 days, 95% CI: −1.77 to 4.93, *p* = 0.35, *I*^2^ = 81%, four studies, 2249 patients, sensitivity analysis: inconsistent) (Figure 5b) [17,19,23,26].

#### 3.2.3. Certainty of Evidence

The certainty of the evidence for the current meta-analysis of observational cohort studies is summarized in Supplemental Table S3. The certainty of evidence was considered low and very low in three (i.e., risk of PO-AKI, infection, and mortality) and four (i.e., risk of bleeding, risk of stroke, ICU stay, hospital stay) outcomes, respectively.

## 4. Discussion

This systematic review and meta-analysis, which aimed at identifying the association between preoperative PNIs and PO-AKI in patients undergoing major surgery with PNI being a categorical (eight studies) or continuous (four studies) variable, showed an association of a low preoperative PNI with an increased risk of PO-AKI. We also found that a low preoperative PNI was related to increased risks of postoperative infection and mortality, as well as a prolonged ICU stay. However, there was no statistical association between preoperative PNI and the risks of postoperative bleeding and stroke.

There are several clinical tools available to assess preoperative nutritional status, including the patient-generated subjective global assessment (PG-SGA), the short-form mininutritional assessment (MNA-SF), the malnutrition universal screening tool (MUST), the nutritional risk screening (NRS-2002), and the nutrition risk index (NRI). It has been shown that these tools are independent predictors of postoperative complications in patients undergoing cardiac surgery [24,25,30,31,32]. However, their complicated scoring systems commonly contribute to interpretation errors [24,25,33]. In contrast, previous studies have shown that PNI could provide an accurate and convenient assessment of preoperative nutritional status [16,24,25].

Despite the first introduction of PNI by Buzby and colleagues as early as 1980 [34], the initially derived method was challenging to apply in clinical practice because of its complexity. Onodera and colleagues [16], after simplifying the PNI equation by incorporating serum albumin level and peripheral blood lymphocyte count, first used the modified approach to predict postoperative morbidity and mortality in patients receiving gastrointestinal surgery in 1984 [16]. After that, PNI has been considered to be one of the most easily measured routine indicators of postoperative outcomes after major surgery. Prior studies have demonstrated an association between a low preoperative PNI and higher recurrence rates after cancer surgery [24]. Furthermore, a low preoperative PNI has independently been reported to predict postoperative mortality and complications in patients undergoing cardiac, lung, and open abdominal surgery [20,23,35].

Previous studies on AKI, which is one of the most frequent postoperative complications, with incidence varying with the type of surgery, mainly focused on cardiac surgery [28]. A systematic review of 35,021 patients undergoing cardiac surgery showed an overall PO-AKI incidence of 25.8% [36]. Another large-scale systematic review enrolled 320,086 patients receiving cardiac surgery and indicated a comparable PO-AKI incidence of 22.3% [37]. In contrast, for noncardiac surgery, a systematic review including 82,514 patients following major abdominal surgery reported a lower PO-AKI incidence of 13.4% [38]. PO-AKI is associated with early and late adverse outcomes, including prolonged hospital stays, increased ICU admissions, the development of chronic kidney disease, and death [4]. Therefore, a greater emphasis has been placed on studying the pathogenesis and perioperative interventions for PO-AKI. It is believed that PO-AKI results from multiple kidney injuries that occur within the preoperative, intraoperative, and postoperative periods [4,12]. The reported mechanisms underlying the development of PO-AKI include renal malperfusion, inflammation, oxidative damage, and exposure to nephrotoxins [4]. Renal hypoperfusion is an important contributor to PO-AKI; previous studies have shown that perioperative hypotension may lead to a pro-inflammatory status with an increase in vasoconstrictive mediators that consequently result in renal tubular ischemia and injury [39]. A variety of potentially nephrotoxic drugs, such as nonsteroid anti-inflammatory drugs (NSAIDs), angiotensin-converting enzyme inhibitors (ACEis), and angiotensin II receptor blockers (ARBs), as well as intravenous contrast media administered in the perioperative setting, are other contributing factors for AKI. In the presence of perioperatively reduced renal blood flow, ACEis, and ARBs may decrease angiotensin II and subsequently lower the glomerular filtration rate (GFR) through loss of efferent arteriolar vasoconstriction [39]. The release of pro-inflammatory cytokines and free radicals in an inflammatory response (e.g., sepsis and systemic inflammation) that frequently occurs following surgery further contributes to renal injury [39].

Despite renal hypoperfusion accounting for a significant majority of PO-AKI, treatments aimed at enhancing renal perfusion or reducing oxidative stress have largely been ineffective. Hence, developing a reliable predictive tool for PO-AKI would be of great value. Our meta-analysis demonstrated a significant correlation between perioperative PNI and PO-AKI in patients undergoing major surgery. Additionally, our results showed that a low PNI was associated with other surgical outcomes, namely postoperative infection, mortality, and a prolonged ICU stay. The results were partially in agreement with those of a previous meta-analysis [18], in which the authors identified PNI as a predictor of survival and postoperative complications after cancer surgery. However, only gastric cancer patients were included in their meta-analysis without further analysis of postoperative complications. To our best knowledge, the current study is the first meta-analysis to systematically explore the relationship between PNI and postoperative complications, especially PO-AKI, in patients undergoing major surgery.

The mechanisms linking a low PNI to adverse surgical outcomes in patients receiving major surgery remain unclear. Taking into account the nutritional and immunological significance of PNI, the following explanations may be plausible. First, PNI may reflect the general physical condition of a patient. Not only has a decreased PNI been shown to indicate a poor general condition and a reduced protein reserve [25], but it has also been reported to be associated with an increased risk of mortality [40]. Second, the multiple roles of albumin, including its ability to maintain oncotic pressure for sustaining renal circulation [10], as well as its antioxidant and anti-inflammatory properties to protect against glomerular and tubular damage [8], may partly explain the correlation between hypoalbuminemia and postoperative complications in patients after a variety of surgical procedures [41]. Recently, a meta-analysis also indicated a correlation between hypoalbuminemia and AKI in hospitalized patients [10]. Third, lymphocytes are an important component of the immune system that is thought to contribute to the onset, proliferation, and recovery of AKI [42]. Preoperative lymphocytopenia has been shown to be associated with postoperative AKI in patients receiving cardiac surgery [15]. Based on those findings, PNI, which is a combination of albumin and lymphocyte levels, could be a reliable predictor of PO-AKI in different clinical settings.

One of the interesting findings of the present meta-analysis was the correlation between a low PNI and increased risks of postoperative gastrointestinal bleeding and stroke, despite a lack of statistical significance probably attributable to a small sample size (i.e., only three studies in each subgroup). Only one previous study indicated that PNI was independently associated with major stroke in patients undergoing carotid artery stenting [43]. Therefore, our findings require further research for elucidation.

The incidence of PO-AKI exhibited considerable variability, ranging from 6.1% to 50.8% in the current meta-analysis. This observed disparity may be partially attributed to differences in the types of surgeries performed, patient characteristics, or methods employed to diagnose PO-AKI. In addition, variations in medical sources and ethnic factors could partially contribute to the discrepancy in incidence rates. For instance, in the context of cardiac surgery, the incidence of PO-AKI significantly varied, with reported rates of 8.3% and 32.2% in Korea and Turkey [22,24], respectively. With the significant variation in PO-AKI incidence, it is important to acknowledge the potential for bias in our results. Future studies focusing on similar clinical settings, surgical procedures, and ethnic populations may provide more robust evidence regarding the association between the PNI and PO-AKI.

Our meta-analysis had several limitations. First, almost all of the studies included were conducted in Asian countries, particularly Korea. Therefore, further studies should include patients of other ethnic backgrounds. Second, the sample size of our analysis was relatively small, with only thirteen studies in total. Third, there was no agreement across the included studies on the precise cutoff value for predicting postoperative complications, particularly AKI. Therefore, more well-designed studies are warranted to verify our findings. Fourth, a study involving 1597 patients requiring intensive care following surgery identified emergency surgery as a risk factor for the development of PO-AKI [44]. However, in the current meta-analysis, only four studies explicitly reported the status of surgeries (i.e., elective), while the remaining studies did not provide this information, leading to uncertainty in the generalizability of our results to emergent surgeries. Fifth, despite the absence of any imposed restrictions on geographical regions in the literature search, our meta-analysis revealed studies exclusively conducted in four countries (i.e., India, China, Turkey, and the Republic of Korea). The lack of studies from other regions (e.g., Europe, Latin America, or the USA) may impose limitations on the generalizability of our findings to diverse ethnic populations. Finally, the heterogeneity in the definitions of PO-AKI among the included studies introduces a potential source of bias, which can make it difficult to draw consistent conclusions. It is important to acknowledge and carefully consider this limitation when interpreting the results.

## 5. Conclusions

In summary, our meta-analysis demonstrated a significant association between a low preoperative PNI and a higher risk of PO-AKI in patients undergoing major surgery. In addition, a low preoperative PNI value correlated with increased risks of postoperative infection and mortality, as well as a prolonged ICU stay. However, no statistical association was noted between a low preoperative PNI and the risks of postoperative bleeding and stroke.

## Figures and Tables

**Table 1:** Characteristics of studies included (*n* = 13).

Studies	Age (Years)	Male (%)	BMI (kg/m^2^)	N	Type of Surgery	PNI Cut-Off Values	AKI Incidence (%)	Country	NOS
Aykut 2022 [21]	61–63	82.2	28–31	455	Cardiac surgery	48	30.9	Turkey	7
Agrawal 2019 [20]	44	57	NA	82	Abdominal surgery ‡	45	9.8	India	9
Dolapoglu 2019 [12]	63–67	75	23	336	Cardiac surgery	NA	26.2	Turkey	7
Gucu 2021 [22]	63–64	59.2	29–30	255	Cardiac surgery	NA	32.2	Turkey	8
Kim 2019 [23]	54	56.8	20.3	132	lung transplantation	41.15	50.8	Korea	8
Lee 2020 [24]	59	57	24.3	374	Cardiac surgery	46.13	8.3	Korea	9
Lin 2021 [25]	53	78.5	24.7	651	Cardiac surgery	41.6	18	China	8
Lu 2020 [26]	55–57	82.9	20–21	41	Lung transplantation	41.15	43.9	China	8
Min 2020 [27]	48–52	71.2	24–25	423	Liver transplantation	NA	12.7	Korea	7
Park 2020 [17]	69 vs. 65	88	22 vs. 24	1011	Lung cancer resection	50	2.5	Korea	7
Sim 2021a [19]	57 vs. 55	80.4	24 vs. 24	1065	Hepatectomy †	44	6.1	Korea	8
Sim 2021b [28]	63	57.9	22.9	817	Open hepatectomy	NA	7.2	Korea	6
Sim 2021c [29]	57–63	61.4	22.9–24.4	3543	Colorectal cancer surgery	NA	10.2	Korea	8

BMI: body mass index; PNI: prognostic nutritional index; AKI: acute kidney injury; NA: not available. ‡ Abdominal surgery consisted of anterior resection of colon, open cholecystectomy, open appendicectomy, and hysterectomy; † open or laparoscopic hepatectomy.

## Data Availability

The original contributions presented in this study are included in this article/Supplementary Material, further inquiries can be directed to the corresponding authors.

## Supplementary Materials

The following supporting information can be downloaded at: https://www.mdpi.com/article/10.3390/nu15132929/s1, Table S1: search strategies for Medline; Table S2: Definition of postoperative acute kidney injury and preoperative creatinine levels; Table S3: summary of findings for the main comparison.

## References

[1] Gameiro J., Fonseca J.A., Neves M., Jorge S., Lopes J.A. (2018). Acute kidney injury in major abdominal surgery: Incidence, risk factors, pathogenesis and outcomes. Ann. Intensive. Care.

[2] Thakar C.V. (2013). Perioperative acute kidney injury. Adv. Chronic Kidney Dis..

[3] Grams M.E., Sang Y., Coresh J., Ballew S., Matsushita K., Molnar M.Z., Szabo Z., Kalantar-Zadeh K., Kovesdy C.P. (2016). Acute Kidney Injury After Major Surgery: A Retrospective Analysis of Veterans Health Administration Data. Am. J. Kidney Dis. Off. J. Natl. Kidney Found..

[4] Boyer N., Eldridge J., Prowle J.R., Forni L.G. (2022). Postoperative AKI. Clin. J. Am. Soc. Nephrol..

[5] Prowle J.R., Forni L.G., Bell M., Chew M.S., Edwards M., Grams M.E., Grocott M.P.W., Liu K.D., McIlroy D., Murray P.T. (2021). Postoperative acute kidney injury in adult non-cardiac surgery: Joint consensus report of the Acute Disease Quality Initiative and PeriOperative Quality Initiative. Nat. Rev. Nephrol..

[6] Billings F.T.T., Lopez M.G., Shaw A.D. (2021). The incidence, risk, presentation, pathophysiology, treatment, and effects of perioperative acute kidney injury. Can. J. Anaesth..

[7] McGuinness S.P., Parke R.L., Bellomo R., Van Haren F.M., Bailey M. (2013). Sodium bicarbonate infusion to reduce cardiac surgery-associated acute kidney injury: A phase II multicenter double-blind randomized controlled trial. Crit. Care Med..

[8] Nipper C.A., Lim K., Riveros C., Hsu E., Ranganathan S., Xu J.Q., Brooks M., Esnaola N., Klaassen Z., Jerath A. (2022). The Association between Serum Albumin and Post-Operative Outcomes among Patients Undergoing Common Surgical Procedures: An Analysis of a Multi-Specialty Surgical Cohort from the National Surgical Quality Improvement Program (NSQIP). J. Clin. Med..

[9] Okugawa Y., Toiyama Y., Yamamoto A., Shigemori T., Ichikawa T., Yin C.Z., Suzuki A., Fujikawa H., Yasuda H., Hiro J. (2020). Lymphocyte-to-C-reactive protein ratio and score are clinically feasible nutrition-inflammation markers of outcome in patients with gastric cancer. Clin. Nutr..

[10] Hansrivijit P., Yarlagadda K., Cheungpasitporn W., Thongprayoon C., Ghahramani N. (2021). Hypoalbuminemia is associated with increased risk of acute kidney injury in hospitalized patients: A meta-analysis. J. Crit. Care.

[11] Ha C.E., Bhagavan N.V. (2013). Novel insights into the pleiotropic effects of human serum albumin in health and disease. Biochim. Et Biophys. Acta.

[12] Dolapoglu A., Avci E., Kiris T., Bugra O. (2019). The predictive value of the prognostic nutritional index for postoperative acute kidney injury in patients undergoing on-pump coronary bypass surgery. J. Cardiothorac. Surg..

[13] Margarson M.P., Soni N. (1998). Serum albumin: Touchstone or totem?. Anaesthesia.

[14] Gibbs J., Cull W., Henderson W., Daley J., Hur K., Khuri S.F. (1999). Preoperative serum albumin level as a predictor of operative mortality and morbidity: Results from the National VA Surgical Risk Study. Arch. Surg..

[15] Aghdaii N., Ferasatkish R., Mohammadzadeh Jouryabi A., Hamidi S.H. (2014). Significance of preoperative total lymphocyte count as a prognostic criterion in adult cardiac surgery. Anesth. Pain Med..

[16] Onodera T., Goseki N., Kosaki G. (1984). Prognostic nutritional index in gastrointestinal surgery of malnourished cancer patients. Nihon Geka Gakkai Zasshi.

[17] Park S., Ahn H.J., Yang M., Kim J.A., Kim J.K., Park S.J. (2020). The prognostic nutritional index and postoperative complications after curative lung cancer resection: A retrospective cohort study. J. Thorac. Cardiovasc. Surg..

[18] Yang Y., Gao P., Song Y., Sun J., Chen X., Zhao J., Ma B., Wang Z. (2016). The prognostic nutritional index is a predictive indicator of prognosis and postoperative complications in gastric cancer: A meta-analysis. Eur. J. Surg. Oncol..

[19] Sim J.H., Kim S.-H., Jun I.-G., Kang S.-J., Kim B., Kim S., Song J.-G. (2021). The association between prognostic nutritional index (PNI) and intraoperative transfusion in patients undergoing hepatectomy for hepatocellular carcinoma: A retrospective cohort study. Cancers.

[20] Agrawal P., Gupta O.P. (2019). An Evaluation of the Prognostic Nutrition Index as a Predictor of Post-Operative Mortality and Morbidity in Patients Undergoing Abdominal Surgery. Int. J. Contemp. Med. Surg. Radiol..

[21] Aykut A., Salman N. (2022). Poor nutritional status and frailty associated with acute kidney injury after cardiac surgery: A retrospective observational study. J. Card. Surgery.

[22] Gucu A., Ozluk O.A., Guvenc O., Sunbul S.A., Engin M. (2021). The Importance of Prognostic Nutritional Index in Predicting Acute Renal Failure After On-Pump Coronary Artery Bypass Operations in Patients with Insulin-Dependent Diabetes Mellitus. Heart Surg. Forum.

[23] Kim C.Y., Kim S.Y., Song J.H., Kim Y.S., Jeong S.J., Lee J.G., Paik H.C., Park M.S. (2019). Usefulness of the preoperative prognostic nutritional index score as a predictor of the outcomes of lung transplantation: A single-institution experience. Clin. Nutr..

[24] Lee S.I., Ko K.P., Choi C.H., Park C.H., Park K.Y., Son K.H. (2020). Does the prognostic nutritional index have a predictive role in the outcomes of adult cardiac surgery?. J. Thorac. Cardiovasc. Surg..

[25] Lin Y., Chen Q., Peng Y., Chen Y., Huang X., Lin L., Zhang X., Chen L.W. (2021). Prognostic nutritional index predicts in-hospital mortality in patients with acute type A aortic dissection. Heart Lung.

[26] Lu K., Li H., Chen Y., Wu B., Zhang J., Huang M., Chen J. (2020). Can the preoperative nutritional risk score be a predictor of the outcomes in critically ill patients of lung transplantation: A retrospective study. Ann. Transl. Med..

[27] Min J.Y., Woo A., Chae M.S., Hong S.H., Park C.S., Choi J.H., Chung H.S. (2020). Predictive Impact of Modified-Prognostic Nutritional Index for Acute Kidney Injury within 1-week after Living Donor Liver Transplantation. Int. J. Med. Sci..

[28] Sim J.H., Bang J.Y., Kim S.H., Kang S.J., Song J.G. (2021). Association of Preoperative Prognostic Nutritional Index and Postoperative Acute Kidney Injury in Patients with Colorectal Cancer Surgery. Nutrients.

[29] Sim J.H., Jun I.G., Moon Y.J., Jeon A.R., Kim S.H., Kim B., Song J.G. (2021). Association of Preoperative Prognostic Nutritional Index and Postoperative Acute Kidney Injury in Patients Who Underwent Hepatectomy for Hepatocellular Carcinoma. J. Pers. Med..

[30] Lomivorotov V.V., Efremov S.M., Boboshko V.A., Nikolaev D.A., Vedernikov P.E., Shilova A.N., Lomivorotov V.N., Karaskov A.M. (2013). Evaluation of nutritional screening tools among patients scheduled for heart valve surgery. J. Heart Valve Dis..

[31] Bauer J., Capra S., Ferguson M. (2002). Use of the scored Patient-Generated Subjective Global Assessment (PG-SGA) as a nutrition assessment tool in patients with cancer. Eur. J. Clin. Nutr..

[32] Kondrup J., Rasmussen H.H., Hamberg O., Stanga Z. (2003). Nutritional risk screening (NRS 2002): A new method based on an analysis of controlled clinical trials. Clin. Nutr..

[33] Stoppe C., Goetzenich A., Whitman G., Ohkuma R., Brown T., Hatzakorzian R., Kristof A., Meybohm P., Mechanick J., Evans A. (2017). Role of nutrition support in adult cardiac surgery: A consensus statement from an International Multidisciplinary Expert Group on Nutrition in Cardiac Surgery. Crit. Care (Lond. Engl.).

[34] Buzby G.P., Mullen J.L., Matthews D.C., Hobbs C.L., Rosato E.F. (1980). Prognostic nutritional index in gastrointestinal surgery. Am. J. Surg..

[35] Bansal N., Magoon R., Dey S., ItiShri I., Walian A., Kohli J.K., Kashav R.C. (2021). Preoperative Combined Adiposity–Nutritional Index Predicts Major aDverse Cardiac and Cerebral Events following Off-pump coronary Artery Revascularization (PANDORA): A retrospective single-center study. J. Card. Crit. Care TSS.

[36] Corredor C., Thomson R., Al-Subaie N. (2016). Long-Term Consequences of Acute Kidney Injury After Cardiac Surgery: A Systematic Review and Meta-Analysis. J. Cardiothor. Vasc..

[37] Hu J.C., Chen R.Y., Liu S.P., Yu X.F., Zou J.Z., Ding X.Q. (2016). Global Incidence and Outcomes of Adult Patients With Acute Kidney Injury After Cardiac Surgery: A Systematic Review and Meta-Analysis. J. Cardiothor. Vasc..

[38] O’Connor M.E., Kirwan C.J., Pearse R.M., Prowle J.R. (2016). Incidence and associations of acute kidney injury after major abdominal surgery. Intens. Care Med..

[39] Bell S., Ross V.C., Zealley K.A., Millar F., Isles C. (2017). Management of post-operative acute kidney injury. QJM-Int. J. Med..

[40] Wada H., Dohi T., Miyauchi K., Jun S., Endo H., Doi S., Konishi H., Naito R., Tsuboi S., Ogita M. (2018). Relationship between the prognostic nutritional index and long-term clinical outcomes in patients with stable coronary artery disease. J. Cardiol..

[41] Rungsakulkij N., Tangtawee P., Suragul W., Muangkaew P., Mingphruedhi S., Aeesoa S. (2019). Correlation of serum albumin and prognostic nutritional index with outcomes following pancreaticoduodenectomy. World J. Clin. Cases.

[42] Weller S., Varrier M., Ostermann M. (2017). Lymphocyte Function in Human Acute Kidney Injury. Nephron.

[43] Ocal L., Kup A., Keskin M., Cersit S., Celik M., Eren H., Gursoy M.O., Ozturkeri B., Ozturk B., Turkmen M.M. (2020). Prognostic significance of pre-procedural prognostic nutritional index in patients with carotid artery stenting. J. Stroke Cereb. Dis..

[44] Abelha F.J., Botelho M., Fernandes V., Barros H. (2009). Determinants of postoperative acute kidney injury. Crit. Care.

