# Supplementary material for: Association of Preoperative Prognostic Nutritional Index with Risk of Postoperative Acute Kidney Injury: A Meta-Analysis of Observational Studies
Source: Nutrients. 2023 Jun 28;15(13):2929. doi: 10.3390/nu15132929 (PMC10346508; doi:10.3390/nu15132929)
Supplement: Supplementary file 1 [file nutrients-15-02929-s001.zip › nutrients-2434803-supplementary.pdf]

Supplemental Table S1. Search strategies for Medline

Database	#	Search syntax
**MEDLINE (Ovid)**	1	("Postoperative" or "Surger*" or "Surgical Procedure*" or "Operative Procedur*s" or "General anesthesia" or "Operation" or "Surgical" or "Cardiac Surgical Procedure*" or "Open heart surger*" or "Valvular heart surger*").mp
	2	exp "Surgical Procedures, Operative"/ or exp "Anesthesia, General"/ or exp "Cardiac Surgical Procedures"/
	3	("Prognostic nutritional index" or "Prognostic Nutritional Indices" or "PNI").mp
	4	("Acute kidney injury" or "AKI" or "Acute renal failure" or "Kidney injury" or "Acute Kidney Insufficiency" or "Kidney Tubular Necrosis" or "Renal Insufficiency").mp
	5	exp "Acute Kidney Injury"/
	7	(1 OR 2) AND (3) AND (4 OR 5)

Supplemental Table S2. Definition of postoperative acute kidney injury and preoperative creatinine levels

Studies	Definition of postoperative AKI	Preoperative creatinine levels
Aykut 2022	KDIGO criteria for stages 1-3	0.95 (0.84-1.1) mg/dL vs. 0.91 (0.81-1.03) mg/dL†
Agrawal 2019	No definition	na
Dolapoglu 2019	AKI was defined as an increase in serum creatinine of at least 0.3 mg/dl or 150– 200% (1.5- to 2-fold) from the baseline.	0.95 (0.79–1.10) mg/dL vs. 0.87 (0.77–0.97) mg/dL†
Gucu 2021	KDIGO criteria for stages 1-3	1.3 (0.6-1.46) mg/dL vs. 0.9 (0.8-1.38) mg/dL†
Kim 2019	Creatinine level to 0.3 mg/dL within 48 h or an increase in the creatinine level by 30% from the baseline value	0.6 (0.4-0.8) mg/dL‡
Lee 2020	AKI was defined as a creatinine elevation of 2-fold the immediate preoperative value or the new requirement for renal replacement therapy	na
Lin 2021	AKI was defined as a creatinine level was 2 times higher than preoperative creatinine or a requirement for renal replacement therapy.	0.98 (0.79-1.4) mg/dL‡
Lu 2020	AKI was described as a rise in the amount of creatinine to 0.3 mg/dL within 48 h or a 50% boost in the level of creatinine from the base value	0.82±0.28 mg/dL vs. 0.76±0.23 mg/dL†
Min 2020	AKI was defined by the AKIN classification; as peak serum creatinine (SCr) 2.0 - 2.9 times (stage II) or ≥ 3.0 times (stage III) baseline levels. Patients who required renal replacement therapy were also classified as having AKI.	na

Park 2020	Acute Kidney Injury Network classification ≥2.	na
Sim 2021a	AKI was defined by Kidney Disease Improving Global Outcomes classification (KDIGO): sCr increased by at least 1.5 times at baseline before surgery within 7 days or sCr increased by 0.3 mg/dL within 48 h	0.84 ±0.17 mg/dL vs. 0.79± 0.18 mg/dL†
Sim 2021b	AKI was defined by Kidney Disease Improving Global Outcomes classification (KDIGO): an increase in sCr ≥ 1.5 times the baseline value, within seven days prior to surgery or increase in sCr by ≥0.3 mg/dL within 48 h	0.82 ± 0.16 mg/dL‡
Sim 2021c	postoperative AKI by Kidney Disease Improving Global Outcomes classification (KDIGO): Increase in sCr by >1.5-fold from preoperative baseline within 7 days or increase in sCr ≥ 0.3 mg/dL within 48 h	0.74-0.83 mg/dL⁋

AKI: Acute kidney injury; na: not available; †patients with acute kidney injury vs. patients without acute kidney injury; ‡overall population; ⁋range of preoperative creatinine levels

Kidney Disease Improving Global Outcomes (KDIGO) classification:

- Stage 1: serum creatinine >1.5-1.9 times, or > 0.3 mg/dl increase> from the basal rate
- Stage 2: serum creatinine 2.0-2.9 times increase from basal rate
- Stage 3: serum creatinine 3 times increase from basal rate or Serum creatinine > 4.0 mg/dl or initiation of renal replacement therapy

Acute Kidney Injury Network (AKIN) classification:

- Stage 1: an increase in serum creatinine of 0.3 mg/dL (or 1.5 to 1.9 times the baseline) within 48 hours, or a urine output less than 0.5 mL/kg/hour for 6 to 12 hours.
- Stage 2: a twofold to threefold increase in serum creatinine, or a urine output less than 0.5 mL/kg/hour for more than 12 hours.

- Stage 3: a threefold increase in serum creatinine within 48 hours, or a serum creatinine level above 4.0 mg/dL, or initiation of renal replacement therapy, or a urine output less than 0.3 mL/kg/hour for more than 24 hours, or anuria (urine output less than 50 mL in 24 hours).

Supplemental Table S3. Summary of findings for the main comparison

Outcomes	Effect (Risk or mean)		Relative effect (95% CI)	№ of participants (studies)	Certainty of the evidence (GRADE)	Comments
	Intervention group	Control group				
PO-AKI risk	206/1268	266/2543	RR 1.65 (1.28 to 2.13)	3811 (7 Studies)	⊕⊕◯◯ Low	-
Infection risk	272/851	220/1440	RR 2.1 (1.67 to 2.64)	2291 (6 Studies)	⊕⊕◯◯ Low	-
Mortality risk	118/788	81/1371	RR 1.93 (1.4 to 2.66)	2159 (5 Studies)	⊕⊕◯◯ Low	-
Bleeding risk	54/489	36/668	RR 2.5 (0.8 to 7.81)	1157 (3 Studies)	⊕◯◯◯ Very Low	a, b
Stroke risk	36/711	30/1325	RR 1.62 (0.96 to 2.7)	2036 (3 Studies)	⊕◯◯◯ Very Low	a
ICU stay	-	-	MD 0.98 (0.15 to 1.81)	2209 (5 Studies)	⊕◯◯◯ Very Low	a, b
Hospital stays	-	-	MD 1.58 (-1.77 to 4.93)	2249 (4 RCTs)	⊕◯◯◯ Very Low	a, b

PO-AKI: postoperative acute kidney injury; ICU: intensive care unit

Comments:
[a]wide 95% CI; [b]The I square is more than 50%.

GRADE Working Group grades of evidence:
High certainty: We are very confident that the true effect lies close to that of the estimate of the effect
Moderate certainty: We are moderately confident in the effect estimate: The true effect is likely to be close to the estimate of the effect, but there is a possibility that it is substantially different
Low certainty: Our confidence in the effect estimate is limited: The true effect may be substantially different from the estimate of the effect
Very low certainty: We have very little confidence in the effect estimate: The true effect is likely to be substantially different from the estimate of effect
